# Development and Initial Validation of the Russian Version of the RAADS-14: A Self-Report Questionnaire to Assess Autistic Traits

**DOI:** 10.3390/ejihpe13110188

**Published:** 2023-11-20

**Authors:** Ivan V. Skorokhodov, Ksenia P. Radygina, Elena Y. Skorokhodova, Svetlana P. Firsova, Galina V. Portnova, Anton A. Varlamov

**Affiliations:** 1 Language and Cognition Laboratory, Pushkin State Russian Language Institute, Moscow 117485, Russia; ivskorokhodov@pushkin.institute (I.V.S.);; 2 Rehabilitation Center for Children with ASD “Our Sunny World”, Moscow 109052, Russia; 3 Foreign Languages Department, Moscow Institute of Physics and Technology, Dolgoprudny 141701, Russia; 4 Faculty of Linguistics, Russian State Social University, Moscow 129226, Russia; 5 Humanitarian Education Center, Volga State University of Technology, Yoshkar-Ola 4240000, Russia; 6 Laboratory of Human Higher Nervous Activity, Institute of Higher Nervous Activity and Neurophysiology of RAS, Moscow 117485, Russia

**Keywords:** autism, self-assessment, high-functioning autism, RAADS-14, autism screening

## Abstract

Autism is a relatively common neurodevelopmental condition that affects social communication and behavior, affecting the overall quality of life. The prevalence of autism is constantly increasing, but timely diagnosis allows for effective treatment. The aim of our research was to construct a Russian version of the RAADS-14, a brief self-report questionnaire originally designed for autism screening, and to perform its initial validation to provide a reference point in developing autism self-assessment tools for the Russian-speaking population. Psychometric properties of the RAADS-14 Rus were evaluated on a sample of 1724 participants, including a general sample of non-psychiatric young adults (n = 794) and adults with clinically established ASD (n = 49); a brief Russian inventory assessing Big Five personality traits (FFQ) was administered to a part of the sample (n = 364) to provide the first assessment of criterion validity. Confirmatory factor analysis of the RAADS-14 Rus confirmed the factor structure of the original Swedish version yielding acceptable fit indices. The discriminating properties were substantially worse than in the original study. The correlations between the RAADS-14 Rus domain scores and the Big Five factors were similar to previously obtained findings. Overall, the results suggest that the RAADS-14 Rus can be used as a screening tool for ASD in adults with proper caution and considering its discriminating properties.

## 1. Introduction

Autism spectrum disorder (ASD) is a range of neurodevelopmental conditions characterized by persisting deficits in social communication and interaction, repetitive and stereotyped behavior, and restricted interests [1]. The prevalence of ASD in the adult population is steadily increasing, it greatly affects the quality of life of adults with ASD and impairs a significant burden on health and social care [2]. Autism in adults with no intellectual impairments is also quite frequent [3], and often underdiagnosed [4], particularly in women [5]. Late identification and general underdiagnosis and misdiagnosis of ASD leads to a problem of ‘a lost generation’ of autistic adults with no intellectual impairments [6]. The comorbidity of ASD and other neuropsychiatric conditions is quite high [7], and differential diagnostics can also pose a problem; therefore, proper assessment of autistic traits and related problems can be crucial for increasing the quality of life of people with ASD [8]. There is a range of self-report questionnaires available for screening of ASD in adults with no intellectual impairments, with the most widely used being the following: the Autism-Spectrum Quotient (AQ, 50 items) [9] and its shorter versions, AQ-10, AQ-20, AQ-J-21, and AQ-S [10]; the Ritvo Asperger and Autism Diagnostic Scale—Revised (RAADS-R, 80 items) [11] and its short version RAADS-14 [8]; the Social Responsiveness Scale, 2nd edition—Adult form (SRS-A or SRS2-A, 65 items) [12]; and the Adult Social Behavior Questionnaire (ASBQ, 44 items) [13]. A systematic review found that stronger evidence of good or satisfactory measurement and diagnostic properties exists for AQ-50, AQ-S, RAADS-R, and RAADS-14 [10].

The RAADS-14 is a 14-item self-report questionnaire specifically designed to be used as a screening tool for ASD and distributed under the CC BY 2.0 Creative Commons License [8]. It was developed on the basis of the longer and more comprehensive RAADS-R, which has demonstrated good overall psychometric properties, test–retest reliability, discriminant and convergent validity, sensitivity, and specificity [11,14,15]. The RAADS-14 was developed using the Swedish version of the RAADS-R [8] and validated on Swedish clinical and non-clinical samples. According to the reported factor structure, the RAADS-14 consists of three factors corresponding to domains of mentalizing deficits, social anxiety, and sensory reactivity. All the items are scored on a four-point Likert scale (ranging from 0 to 3) indicating the duration of each symptom (3 = ‘true now and when I was young’, 2 = ‘true only now’, 1 = ‘true only when I was younger than 16’, and 0 = ‘never true’). Item scores are summed to produce the total score and the subscale scores. The recommended cut-off of 14 for the original English version yielded an excellent sensitivity of 0.97 and specificity of 0.95 over the non-psychiatric population. The gender differences in the original validation study were significant but of moderate effect size: neurotypical males scored somewhat higher than females in the mentalizing deficits and social anxiety domains, while females scored higher than males in the sensory reactivity domain.

The RAADS-14 is short and easy to administer and has already demonstrated the potential to distinguish efficiently between ASD and other neuropsychiatric conditions in the adult population.

The situation with clinical diagnostics of ASD in the adult population in Russia remains very problematic but is changing for the better, with underdiagnosis being the major issue. According to the official statistical data by Rosstat (Russian statistical agency), in 2016 there were only 96 adults officially diagnosed as having ASD (less than 0.001% of the adult population) [16], which is at least 1000 times less than the estimates based on European cohort statistics. This huge disagreement reveals the fact that it was virtually impossible to obtain an official diagnosis of ASD for high-functioning adolescents and adults until recently for two major reasons. The first reason is related to the underdiagnosis of ASD in childhood from 1970 to the 2010s, which was less pronounced than for adults but still quite prominent: as of 1999, the officially recognized ratio was 1 in 385 [17]. For children without intellectual impairments, the diagnosis was almost never officially established, often in order to help the parents avoid the stigma [18]. The second reason is an almost routine obligatory change in the official clinical diagnosis from ASD to some other neuropsychiatry condition performed when a person reaches adolescence or adulthood, most commonly, to schizoaffective disorder and/or mental retardation; this clinical practice was officially reproved only on 30 June 2014 [19]. Due to the efforts of governmental and non-governmental institutions, autism professionals, the parental community, and the autism community, it is now widely recognized that ASD in adults is currently underdiagnosed in Russia. Implementing the diagnostic routines will require some time and is hindered by the lack of diagnostic tools and trained professionals. The Russian version of ADOS-2 was published only in 2016 [20,21] and its adult module is still undergoing validation.

In this situation, the need for self-assessment tools in the autistic community has long been perceived as high. Despite that, to the best of our knowledge, no self-assessment tools for evaluating autistic traits and for screening ASD in the adult population or adolescents have been officially developed, adapted, and validated on Russian-speaking samples. Several attempts were made by members of the autistic community to provide Russian versions of internationally recognized questionnaires, even if just for a rough reference; the largest collection of unofficial translations (including AQ, RAADS-R, Aspie Quiz, and The Broad Autism Phenotype Test) is presented on a website developed and maintained by members of the Russian autism community that provides information related to autism and the problems of people with ASD [22]. There are multiple anecdotal pieces of evidence that indicate that most of these questionnaires used with cut-offs established for European samples tend to overestimate the prominence of autistic traits, but no data on this issue have been officially reported.

The complete lack of ASD diagnostic tools and inventories assessing autistic traits validated on Russian-speaking samples significantly limits the options to assess the criterion validity of any emerging ASD screening questionnaire. In order to obtain additional circumstantial evidence, special efforts could be made to evaluate the questionnaire’s discriminant validity comparing it to other personality trait inventories. The Big Five personality model is probably the most well-established general personality construct [23], which was found to strongly relate to autism traits in a large number of studies, consistently revealing moderate positive correlations of ASD scores to neuroticism, and weak to moderate negative correlations to all the other Big Five factors [24]. A study utilizing multivariate approaches to explore the relations between IPIP-NEO-120 and RAADS-R scores found that Big Five personality factors accounted for 70% of the variance in autism trait scores [25]. Thus, as the Big Five relationship to the original RAADS-R is known, replication of those findings with a validated Russian Big Five questionnaire and RAADS-14 translation appropriately would be another evidence of a close relationship between the English and the Russian versions of RAADS-14.

The above-mentioned study was based on the pilot study by the same group presented at the INSAR virtual conference in 2020 [26]. The results of the current study differ in the number of participants (in particular, the ASD group was expanded); it minorly affected the factor composition and slightly increased the discriminative power.

The general aim of our research was to construct and validate a Russian version of the RAADS-14 (the RAADS-14 Rus) to provide a reference point in developing the body of instruments for assessing autistic traits in the Russian-speaking adult population. This measure was chosen due to its good psychometric characteristics, discriminative properties, and simplicity of use. The study aims to assess the reliability of the RAADS-14 Rus and its factor structure and to provide the initial estimates of the questionnaire’s validity, diagnostic properties, and potential for use as a screening tool for ASD in adults with no intellectual impairments. We have also formulated three specific research hypotheses:

1. The RAADS-14 Rus factor structure would be equivalent or similar to the factor structure of the original RAADS-14;
2. The distribution of the RAADS-14 scores could be different for the general Russian-speaking population, producing greater mean total score values and yielding potentially higher cut-off scores to provide reasonable specificity of the questionnaire;
3. The RAADS-14 Rus total scores and subscale scores will have weak to moderate correlations to the scores of the Big Five personality factors, in particular, positive to neuroticism and negative to extraversion, consistent with the previous studies.

## 2. Materials and Methods

### 2.1. Materials

The translation was conducted according to the current best practices in the field [27]. The original 14 items of the RAADS-14, as published in [8], were translated from English into Russian independently by a team of three certified translators, all of whom had prior experience with ASD-related problems of at least 2 years. A consensus version was composed collegially by the three translators, an impartial editor, and a member of the ASD community with more than 5 years of consulting experience with ASD-related problems. Back-translation performed independently by two English native speakers unfamiliar with the original revealed no meaningful disagreement with the original version. As in the original RAADS-14, all the items were scored on a four-point Likert scale (ranging from 0 to 3) indicating the duration of each symptom (3 = ‘true now and when I was young’, 2 = ‘true only now’, 1 = ‘true only when I was younger than 16’, and 0 = ‘never true’). The item scores were summed to produce the total score (for general total score statistics by samples see Table 1). The Russian version of the form is provided in the Supplementary Materials (Document S1). To assess the Big Five personality factors, we used a Russian version of the NEO-FFI [28]. This questionnaire is reasonably brief (60 items) and its factor structure has been consistently replicated for different Russian-speaking samples [29,30]. The questionnaire has 60 items (12 items for each scale: openness to experience, conscientiousness, extraversion, agreeableness, and neuroticism) scored on a five-point Likert scale (from 1, ‘completely disagree’, to 5, ‘completely agree’).

### 2.2. Participants and Procedure

A total of 1724 adults agreed to voluntarily participate in the study. The data were collected for three different samples:

- Sample 1: Non-psychiatric control sample of young adults (college and vocational school students, total n = 849) who volunteered to participate for research credits. The recruitment was performed in 4 educational institutions in Moscow (n = 522) and 5 educational institutions in other Russian regions (n = 327); the consent rate was above 0.7. A part of the participants (n = 652) used paper and pen versions while other participants used web-based survey forms and completed them in class settings. A total of 57 participants from Sample 1 failed to provide the answers to all the questions and were excluded from further analyses, reducing the number of accepted participants for Sample 1 to 794. Three participants did not report their gender; therefore, the total number of participants from the sample is greater than the sum of female (n = 504) and male (n = 287) participants.
- Sample 2: A sample was recruited using snowball sampling by targeting autism-related communities to ensure a higher-than-normal ratio of adults with ASD (total n = 509). To provide the initial estimates of the sensitivity of the RAADS-14 Rus in this study, we relied on self-reported ASD diagnosis, reportedly established in clinical settings (n = 49) similar to another recent study assessing the RAADS-14 properties for a different national sample [31]; for a smaller subset of the participants that gave their consent (n = 13), it was further verified using ADOS-2 conducted by a certified specialist. All the participants in this sample used web-based survey forms and completed them at their convenience.
- Sample 3: An additional web-based sample was recruited using snowball sampling (total n = 364) to provide the data for evaluating possible relations between the autism traits measured using the RAADS-14 Rus and the Big Five personality traits. All the participants in this sample used web-based survey forms and completed them at their convenience.

After all the exclusions, the data from a total of 1667 participants were used for further analyses. The data collection was anonymous for all the participants but the participants from samples 2 and 3 were requested to provide their emails (this was optional and the emails were used for the sole purpose of providing feedback with the participant’s personal RAADS-14 Rus scores and general follow-up for the study including a concise half-page outline of the study in lay language). All the participants provided their informed consent before completing the questionnaire. All the aspects of the data collection and treatment reported in the present article were approved by the Pushkin Institute research ethics committee. For detailed age and gender statistics for all the samples, see Table 1. 

### 2.3. Data Analysis

#### 2.3.1. Reliability and the Factor Structure

To evaluate the reliability and the factor structure of the RAADS-14 Rus, we pooled the participants from all the samples resulting in a total of 1667 participants. Cronbach’s α was used to assess the reliability of the scale. In order to assess whether the factor structure of the RAADS-14 Rus was equivalent to the factor structure of the original version, confirmatory factor analysis (CFA) was performed using a single-order three-factor model reported in the original validation study, and a default one-factor model was used as a comparison model. A weighted least-squares with mean and variance (WLSMV) procedure was used to estimate the model. The goodness of model fit was tested by calculating the CFI (comparative fit index) and the root-mean-square-error of approximation (RMSEA). 

#### 2.3.2. Demographics and Correlation Analyses

Age and gender effects were assessed separately for all the samples due to prominent differences in the demographics and clinical characteristics of the samples. A Kolmogorov–Smirnov test was performed for total RAADS-14 Rus for the pooled sample revealing that the data do not have a normal distribution (n = 1667, Mean = 17.42, SD = 9.96, D = 0.07, *p* < 0.001). Due to the non-normal nature of the data, all the group-related differences were assessed with Kruskal–Wallis ANOVA and Mann–Whitney *U* tests. Spearman’s rank-order correlations were used for subscale cross-correlations and correlations with the Big Five factor scores. 

#### 2.3.3. Screening Properties

The discriminatory power was assessed using a receiver operating characteristic (ROC) curve, estimating sensitivity (the rate of true positives for the ASD sample), specificity (1 minus the rate of false positives for the non-psychiatric control sample), and the area under the curve (AUC) as a measure of the discriminatory power (an AUC greater than 0.7 is generally considered satisfactory) [32].

#### 2.3.4. Statistical Analysis

All the statistical analyses were performed using Statistica 10, IBM SPSS Statistics 23, and Mplus 8.2 software [33]. Bonferroni corrections for multiple comparisons were used where appropriate. We conducted confirmatory factor analysis for categorical variables on the second subset (n = 762) of the total sample using Mplus [33]. A weighted least-squares with mean and variance adjustment procedure was used as well. The goodness of model fit was also tested using RMSEA and CFI. All the samples (three sub-samples and a resulting pooled sample) and applied analyses are listed below in Table 2.

## 3. Results

### 3.1. Reliability and the Factor Structure

Cronbach’s α for the 14 items of the RAADS-14 Rus was 0.839, demonstrating good reliability of the questionnaire with an average inter-item correlation of 0.272.

**Figure 1:**
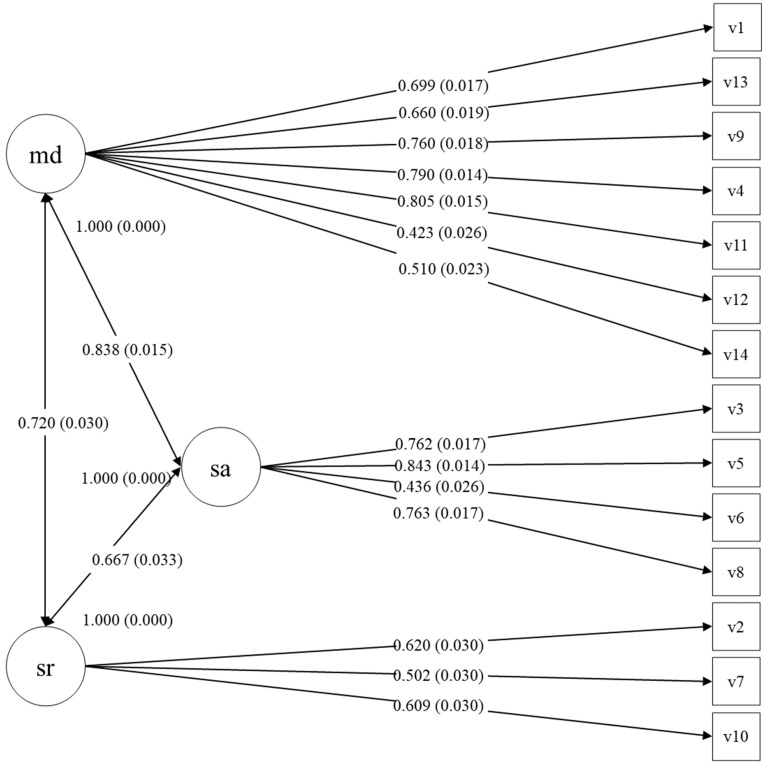
The path diagram for the RAADS-14 Rus with factor loadings and covariances. md—mentalizing deficits, sa—social anxiety, and sr—sensory reactivity.

The CFA performed for a three-factor model reflecting subscale item composition of the original RAADS-14 (Figure 1) revealed acceptable fit indices (RMSEA = 0.067, CFI = 0.954, df = 91, χ^2^ =12,076.025, *p* < 0.001). The CFI demonstrated a good fit [34] and the RMSEA demonstrated a moderately good fit [35]. All the factor loads were above 0.42. The data suggest that the factor structure of the RAADS-14 Rus is very similar to the factor structure of the original Swedish version; therefore, the three domains (mentalizing deficits, social anxiety, and sensory reactivity) of the RAADS-14 RUS were scored as in the original version.

### 3.2. Distribution of Scores and Discriminative Properties

**Figure 2:**
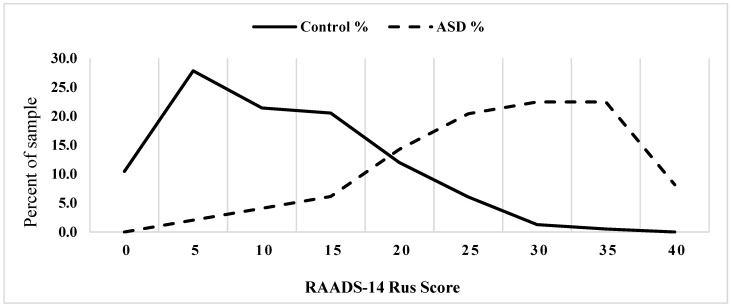
The distribution of the RAADS-14 Rus total scores for non-psychiatric young adults (bold line) and ASD participants (dashed line).

The distributions of total RAADS-14 Rus scores in non-psychiatric young adults (n = 794, mean = 13.9, SD = 7.5, median = 13) and ASD participants (n = 49, mean = 29.3, SD = 8.7, median = 30.5) are shown at Figure 2. The score distribution in non-psychiatric young adults, as expected, was skewed to the left, while the score distributions for self-diagnosed and clinically diagnosed adults with ASD were very similar and were skewed to the right, with no prominent ceiling effects observed for any of the samples. An additional comparison revealed that the RAADS-14 Rus scores of ASD participants with ADOS-2 verified diagnosis were slightly higher (n = 13, mean = 33.2, SD = 7.1, median = 35.0) than the scores of the rest of the ASD participants but the difference was not statistically significant (U = 170.5, *p* = 0.150). The distribution of scores for the ASD participants was similar to the data obtained with the RAADS-14 for the Swedish ASD population [8]. The distribution of the RAADS-14 Rus scores for the non-psychiatric adults was less extremely skewed to the left compared with the Swedish data, producing much greater mean and median scores (13.9 and 13 vs. 3.9 and 3), confirming our prediction that mean RAADS-14 Rus scores are expected to be higher than the original scores. The comparison of the domain scores between the non-psychiatric participants (Sample 1 n = 794) and the participants with clinically established ASD diagnosis (n = 49) revealed very robust effects (U_MD_ = 4773, *p* < 0.001; U_SR_ = 8404.5. *p* < 0.001; U_SA_ = 3685.5, *p* < 0.001), indicating that the ASD participants scored higher on each domain compared with the non-psychiatric participants.

**Figure 3:**
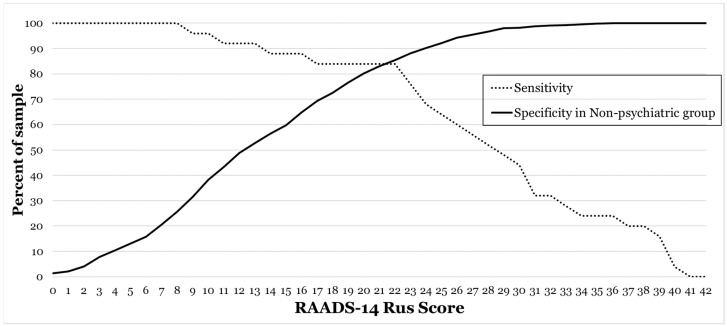
Operating characteristics of the RAADS-14 Rus. Sensitivity and specificity for various threshold scores for individuals with autism spectrum disorder (ASD) (dashed line) compared with non-psychiatric individuals (bold line).

An ROC curve comparing the results of non-psychiatric young adults and participants with ASD diagnosis yielded an AUC of 0.92 (Figure S1), evidencing reasonably good discriminative properties of the RAADS-14 Rus. The operating characteristics of the RAADS-14 Rus are shown in Figure 3. The originally suggested cut-off of 14 points and above would yield a sensitivity of 93.9% and a specificity of 56.4%; a cut-off of 22 points and above would yield a sensitivity of 85.7% and a specificity of 85.4%.

### 3.3. Age and Gender Differences

Considering the large difference in mean age and the RAADS-14 Rus scores between the samples, the assessment of possible relations between age and the RAADS-14 Rus total scores was performed separately for all the samples and, additionally, for the participants with ASD. Weak but significant negative correlations were observed for Sample 1 (n = 793, r = −0.017, *p* = 0.629), Sample 2 (n = 509, r = −0.32, *p* > 0.001), and Sample 3 (n = 364, r = −0.1, *p* = 0.058). This correlation was not significant for the ASD subgroup due to the smaller sample size (n = 49, r = −0.13, *p* = 0.388). 

The gender differences were also assessed separately for all the samples and the participants with ASD. The participants who reported their gender as ‘other’ (Sample 2 n = 20; Sample 3 n = 16) were characterized by significantly higher RAADS-14 Rus total scores than the participants stating their gender as both male or female for Sample 2 (H (2, N = 509) = 3.92, *p* > 0.001) and for Sample 3 (H (2, N = 364) = 10.4. *p* = 0.006) (see Table 1). There were no significant differences between the males and the females in the RAADS-14 Rus total scores for any of the samples, nor for the ASD subgroups.

The RAADS-14 Rus domain score comparison for males and females in the non-psychiatric population revealed that similar to the RAADS-14 validation study, females scored higher than males for the sensory reactivity domain (U = 5815.5, *p* < 0.001. females: mean = 4.12, SD = 2.35; males: mean = 3.29, SD = 2.44). No significant differences were found between males and females for the mentalizing deficits and social anxiety domains of the RAADS-14 Rus (U = 71,851,5, *p* = 0.878 and U = 68,964,5, *p* = 0.272).

### 3.4. Correlation with Big Five Personality Traits

The correlations of the RAADS-14 Rus total scores and domain scores with the Big Five personality factors are given in Table 3. The total score yielded moderate positive correlations with neuroticism (0.45), moderate negative correlations with extraversion (−0.49), and weak but significant negative correlations with the other Big Five factors. Social mentalizing and social anxiety domains each yielded similar correlation patterns, while the sensory and cognitive perception domains had weaker correlations with the Big Five facets reaching significance only for neuroticism and extraversion.

## 4. Discussion

The aim of this study was to develop a Russian version of the RAADS-14 (the RAADS-14 Rus) and to assess its reliability, factor structure, validity, and discriminative properties. The RAADS-14 Rus has exactly the same item composition as the original version; confirmatory factor analysis (CFA) revealed that all the model fit indices were reasonably good, suggesting that the factor structure of the Russian version is equivalent to the original version [8], confirming our first research hypothesis. There was a very robust difference in the scores of all the RAADS-14 Rus screening domains between the non-psychiatric population and the ASD sample, further confirming the construct validity of the RAADS-14 Rus. 

The original RAADS-14 was composed of the items yielding the highest discriminative properties for ASD diagnosis; owing to this approach, the questionnaire was characterized by excellent discriminative properties at distinguishing between ASD participants and non-psychiatric controls (AUC = 0.99) and by good discrimination between ASD and other neuropsychiatric conditions. The total RAADS-14 Rus score has good discriminative properties for distinguishing between ASD participants and the general population control sample (AUC of 0.92), which is still not nearly as good as in the original study. This difference can be best attributed to the fact that mean RAADS-14 scores were much higher for the Russian sample than for the original Swedish sample, particularly for non-psychiatric controls. At the moment we would recommend using a provisional cut-off of 22 points and above. This cut-off yields a sensitivity of 85.7% and a specificity of 85.4%. While taken per se these discriminative properties may seem operational, it should be noted that using the RAADS-14 Rus as a screening tool would yield a low positive predictive value, given the estimated prevalence of high-functioning forms of ASD. Using an official prevalence estimate of high-functioning autism of 1/200 for Russian samples [17], the positive predictive value would be as low as 0.029 (one correctly identified autistic person for 32 non-autistic individuals erroneously identified as autistic). Still, at the moment, with no other validated ASD self-assessment tools available for the Russian-speaking population, we suggest that one can use RAADS-14 Rus for screening if all the above-mentioned considerations are taken into account. Further research is sought to develop more sensitive autism screening tools specifically for Russian-speaking samples.

While this paper was in preparation, the results of another study assessing the RAADS-14 properties for a New Zealand national sample were published [31]. The two studies are similar in their purpose and design, and there are notable similarities in the results as well. In both studies, the mean RAADS-14 scores for non-ASD groups were much higher than in the original study (Russia 13.9, New Zealand 9.85 vs. Sweden 3.9); this, combined with other possible factors, led to substantial worsening of the discriminative properties of the questionnaire. The mean RAADS-14 scores were the highest for the Russian study; this confirms our second research hypothesis originated from prior anecdotal evidence that pilot non-validated versions of autism assessment self-report questionnaires produce higher scores for Russian samples than for Western European and American samples. The underlying reasons for this effect are yet unclear; several factors may contribute but at this moment we would suggest that this difference is to be largely attributed to culture-specific biases in self-reporting and speaking about one’s problems and social competence rather than to elevated autistic traits in the Russian neurotypical population. Despite that, the RAADS-14 Rus performed somewhat better than the New Zealand version of the RAADS-14, producing better model fit indices and somewhat better sensitivity and specificity for the author-suggested cut-offs. 

The RAADS-14 Rus total score revealed a weak but significant negative correlation with age for the non-psychiatric young adult sample, consistent with the previous data indicating that different facets of autistic traits in adults tend to somewhat decrease with age [36,37]. The female-to-male differences in the RAADS-14 Rus scores were quite minor and generally similar to data from the original RAADS-14 validation study collected for Swedish samples [8]. In our study, a significant part of subjects in Samples 2 and 3 indicated their gender as ‘other’ or non-binary (total n = 36). The non-binary participants scored significantly higher than the males and females; this also corresponds to the prior findings in other national samples [38,39,40].

The correlations of the Big Five personality traits with the total RAADS-14 Rus score, taken as a general measure of autistic traits, were highly consistent with the earlier findings [24], revealing moderate positive correlations with neuroticism, moderate negative correlation with extraversion, and weaker negative correlations with agreeableness, openness to experience, and conscientiousness, similar to a study using RAADS-R and IPIP-NEO-120 [25]. These results support good discriminative validity of the RAADS-14 Rus and confirm our third research hypothesis that the character of the relation between autistic traits, as measured using the RAADS-14 Rus, and the Big Five personality traits for the Russian sample is similar to such relation for other national populations. It provides evidence of criterion validity of the RAADS-14 Rus, as it correlates with a Big Five version validated for the Russian population in the same manner as the original RAADS-14 does with an English version, which means they measure similar traits.

## 5. Conclusions

The main limitations of this study stem from the lack of validated ASD assessment tools currently available in the Russian language and from extremely high estimates of underdiagnosis of ASD in Russian adults with no intellectual impairments. Due to our limited options to assess the concurrent validity of the RAADS-14 Rus and the limited size of the ASD sample in this study (particularly due to the small number of participants with ADOS-verified diagnosis), all the estimates of the discriminative properties of the RAADS-14 Rus should be interpreted strictly as preliminary. These findings require further validation on larger clinical and non-clinical samples with a more controlled and straightforward recruiting and selection process.

However, considering that (a) to the best of our knowledge, the RAADS-14 Rus is the first self-assessment inventory addressing autistic traits in adults with no intellectual impairments and (b) the non-psychiatric sample of young adults is relatively large and represents several Russian regions, the data were collected with high consent rate and can be considered a satisfactory initial approximation of the age cohort, at least for larger cities [41]. Therefore, the RAADS-14 Rus can become one of the first stepping stones in establishing a comprehensive system of ASD assessment and diagnostics for the adult Russian-speaking population.

Further efforts should be made to develop tools with better screening sensitivity; to ensure this, the new screening instruments could be devised using larger initial pools of ASD-related items and an item selection process centered around single-item discriminatory power, similar to the process used for developing the original RAADS-14.

## Figures and Tables

**Table 1:** General characteristics of the study samples.

Sample	Gender Distribution	Age Mean	Age SD	Mean RAADS-14 Score	RAADS-14 Score SD
Sample 1	Females (n = 504)	20.3	5.4	14.3	7.4
	Males (n = 287)	19.9	3.8	13.4	7.7
	Total (n = 794) ^1^	2.1	4.9	13.9	7.5
Sample 2	Females (n = 331)	35.2	1.0	21.5	11.8
	Males (n = 128)	36.3	11.8	21.3	1.7
	Other (n = 50)	27.1	8.6	3.92	8.14
	Total (n = 509)	34.7	1.7	22.36	11.57
Sample 3	Females (n = 263)	31.7	1.1	18.5	9.2
	Males (n = 85)	32.4	9.5	16.1	8.2
	Other (n = 16)	33.0	9.0	24.3	9.0
	Total (n = 364)	31.9	9.9	18.22	9.12

^1^ Three participants refused to report their gender.

**Table 2:** List of samples and analyses applied to them.

Sample	N Participants	Age and Gender Correlations	Sensitivity/ Specificity	Reliability	Factor Structure	Big 5 Correlation
Sample 1	794	✓	-	-	-	-
Sample 2	509	✓	-	-	-	-
Sample 3	364	✓	-	-	-	✓
Total sample	1667	-	✓	✓	✓	-

**Table 3:** The correlations (Spearman’s rho) between the RAADS-14 Rus total scores and subscores and the Big Five personality factors.

Big Five Traits	RAADS-14 Rus Total	*p*	Mentalizing Deficits	*p*	Sensory Reactivity	*p*	Social Anxiety	*p*
Neuroticism (n = 325)	0.45	<0.0001	0.34	<0.0001	0.24	<0.0001	0.42	<0.0001
Extraversion (n = 325)	−0.46	<0.0001	−0.32	<0.0001	−0.19	0.0007	−0.53	<0.0001
Openness to experience (n = 325)	−0.13	0.0178	−0.18	0.0011	0.02	0.7324	−0.11	0.0549
Agreeableness (n = 325)	−0.17	0.0023	−0.15	0.0082	−0.06	0.2973	−0.18	0.0012
Conscientiousness (n = 325)	−0.23	<0.0001	−0.16	0.0042	−0.09	0.1176	−0.25	<0.0001

## Data Availability

The datasets created and analyzed during the current study are available from the corresponding author upon reasonable request.

## Supplementary Materials

The following supporting information can be downloaded at: https://www.mdpi.com/article/10.3390/ejihpe13110188/s1, Document S1: Russian version of RAADS-14 Rus and English version of RAADS-14, Document S2: Alternative models, Figure S1: ROC curve.
