# Supplementary material for: Development and Initial Validation of the Russian Version of the RAADS-14: A Self-Report Questionnaire to Assess Autistic Traits
Source: Eur J Investig Health Psychol Educ. 2023 Nov 20;13(11):2724–35. doi: 10.3390/ejihpe13110188 (PMC10670239; doi:10.3390/ejihpe13110188)
Supplement: Supplementary file 1 [file ejihpe-13-00188-s001.zip › S1. RAADS-14 form.pdf]

RAADS-14 Rus

**ФИО:**	**Дата:**

Пожалуйста, оцените, насколько верно каждое из утверждений наиболее точно описывает вас в настоящее время и в детстве (до 16 лет), и поставьте отметку в соответствующей графе. Пожалуйста, ограничьте свой выбор одним ответом для каждого пункта.

	Переживания и индивидуальные особенности, которые могут вас характеризовать	Верно и сейчас, и в детстве	Верно только сейчас	Было верно только для возраста до 16 лет	Неверно и никогда не было верно
1.	Во время разговора с другими людьми мне сложно понять, что они чувствуют	☐	☐	☐	☐
2.	Мне очень неприятно ощущать прикосновение некоторых обычных материалов к своей коже, хотя других они, похоже, не раздражают	☐	☐	☐	☐
3.	Мне очень сложно действовать и работать в коллективах	☐	☐	☐	☐
4.	Мне сложно понять, что от меня ожидают другие люди	☐	☐	☐	☐
5.	Я часто не знаю, как вести себя при взаимодействии с другими людьми	☐	☐	☐	☐
6.	Я могу поболтать о пустяках с другими людьми	☐	☐	☐	☐
7.	Когда я чувствую, что перегружен ощущениями, мне нужно уединиться, чтобы приглушить их.	☐	☐	☐	☐
8.	Как находить друзей и общаться – загадка для меня	☐	☐	☐	☐
9.	Когда я разговариваю с кем-то, мне трудно понять, когда моя очередь говорить или слушать	☐	☐	☐	☐
10.	Иногда мне приходится затыкать уши, чтобы приглушить нестерпимый шум (например, звук пылесоса или слишком громкий или многословный разговор)	☐	☐	☐	☐
11.	Мне бывает очень сложно понять выражение лица, позу и значение жестов собеседника.	☐	☐	☐	☐
12.	Я концентрируюсь на деталях, а не на общей идее	☐	☐	☐	☐
13.	Я воспринимаю сказанное слишком буквально, поэтому часто упускаю, что пытается сказать собеседник	☐	☐	☐	☐
14.	Меня очень расстраивает, когда мне приходится изменять привычный образ действий	☐	☐	☐	☐

Подсчет баллов

Большинство вопросов имеют прямое кодирование.
Верно и сейчас, и в детстве = 3, Верно только сейчас = 2, Было верно только для возраста до 16 лет = 1, Неверно и никогда не было верно = 0

В вопросах, отмеченных буквой R, следует использовать обратное кодирование (Верно и сейчас, и в детстве = 0, Верно только сейчас = 1, Было верно только для возраста до 16 лет = 2, Неверно и никогда не было верно = 3).

Для оценки каждой из шкал сложите результаты соответствующих вопросов:
Социальная ментализация (СМ) (7 пунктов, минимальное значение 0, максимальное значение 21): 1, 4, 9, 11, 12, 13, 14
Сложности социального взаимодействия (ССВ) (4 пунктов, минимальное значение 0, максимальное значение 12): 3, 5, 6R, 8
Сенсорное восприятие и когнитивный стиль (СВиКС) (3 пункта, минимальное значение 0, максимальное значение 9): 2, 7, 10,

Итоговая оценка получается сложением результатов по всем трём шкалам и может использоваться при скрининге расстройств аутистического спектра (минимально возможное значение 0 баллов, максимально возможное значение 42 балла).

Интерпретация результатов:

От 0 до 16 баллов — низкая выраженность аутистических характеристик и очень низкий риск наличия РАС;

От 17 до 21 балла — умеренная выраженность аутистических характеристик, низкий риск наличия РАС;

От 22 до 29 баллов — высокая выраженность аутистических характеристик, значительный риск наличия РАС;

От 30 до 42 баллов — очень высокая выраженность аутистических характеристик, высокий риск наличия РАС.

RAADS-14 Screen

Name:	Patient ID:
Date:	Clinician:

Please choose one of the following alternatives:
This is true or describes <u>me now and when I was young</u>.
This was true or describes me <u>only now</u> (refers to skills acquired).
This was true <u>only when I was young</u> (16 years or younger).
This was <u>never true and never described me.</u>
Please answer the questions according to what is true for *you*. Check only one column per statement!

	Some life experiences and personality characteristics that may apply to you	True now and when I was young	True only now	True only when I was younger than 16	Never true
1.	It is difficult for me to understand how other people are feeling when we are talking	☐	☐	☐	☐
2.	Some ordinary textures that do not bother others feel very offensive when they touch my skin	☐	☐	☐	☐
3.	It is very difficult for me to work and function in groups	☐	☐	☐	☐
4.	It is difficult to figure out what other people expect of me	☐	☐	☐	☐
5.	I often don’t know how to act in social situations	☐	☐	☐	☐
6.*	I can chat and make small talk with people	☐	☐	☐	☐
7.	When I feel overwhelmed by my senses, I have to isolate myself to shut them down	☐	☐	☐	☐
8.	How to make friends and socialize is a mystery to me	☐	☐	☐	☐
9.	When talking to someone, I have a hard time telling when it is my turn to talk or to listen	☐	☐	☐	☐
10.	Sometimes I have to cover my ears to block out painful noises (like vacuum cleaners or people talking too much or too loudly)	☐	☐	☐	☐
11.	It can be very hard to read someone’s face, hand, and body movements when we are talking	☐	☐	☐	☐
12.	I focus on details rather than the overall idea	☐	☐	☐	☐
13.	I take things too literally, so I often miss what people are trying to say	☐	☐	☐	☐
14.	I get extremely upset when the way I like to do things is suddenly changed	☐	☐	☐	☐

RAADS-14 Screen

Scoring:

- All items (except item 6 which is reversed and therefore marked with*) range from 3 to 0.
- The score ranges from a minimum of 0 to a maximum of 42.

RAADS-14 Screen contains three subdomains:

• Mentalizing deficits: items 1, 4, 9, 11, 12, 13, 14	(explaining 32.5% of the variance)
• Social anxiety: items 3,5,6,8	(explaining 8.5% of the variance)
• Sensory reactivity: items 2,7,10	(explaining 6.4% of the variance)

Median score for different populations:

- **32** in 135 adults with normal intelligence diagnosed with autism spectrum disorder (ASD).
- **15** in 344 adults with normal intelligence diagnosed with attention deficit hyperactivity disorder (ADHD).
- **11** in the collapsed group of 164 adults with other psychiatric disorders, specified as psychotic disorder (n=30), mood disorder (n=59), anxiety disorder or OCD (n=67) and borderline personality disorder (n=39). The sums of reported diagnoses exceed the number of patients in the group because of co-occurring diagnoses.
- **3** in 590 non-psychiatric controls

A cut-off score of 14 or above reached a sensitivity of 97% and a specificity of:

- 46% for the ADHD group.
- 64% for the other psychiatric disorders group (described above).
- 95% for the non-psychiatric controls.

The discriminatory power of the first five items in the RAADS-14 Screen:

- A cut-off point of 4 or greater from a maximum of 15 points yielded a sensitivity of 93% and a specificity of 45% in the ADHD sample, 49% in the other psychiatric disorders group, and 90% in the non-psychiatric sample.

RAADS-14-Screen is an abridged version of Ritvo Autism and Asperger Diagnostic Scale-Revised (RAADS-R). Cite: Eriksson JM, Andersen MJ, Bejerot S. RAADS-14 Screen: validity of a screening tool for Autism Spectrum Disorder in an adult psychiatric population. Molecular Autism 2013; 4:49.
