# Supplementary material for: Development and Initial Validation of the Russian Version of the RAADS-14: A Self-Report Questionnaire to Assess Autistic Traits
Source: Eur J Investig Health Psychol Educ. 2023 Nov 20;13(11):2724–35. doi: 10.3390/ejihpe13110188 (PMC10670239; doi:10.3390/ejihpe13110188)
Supplement: Supplementary file 1 [file ejihpe-13-00188-s001.zip › S2.Alternative models.pdf]

Three factor model without covariances considered (Model 1)

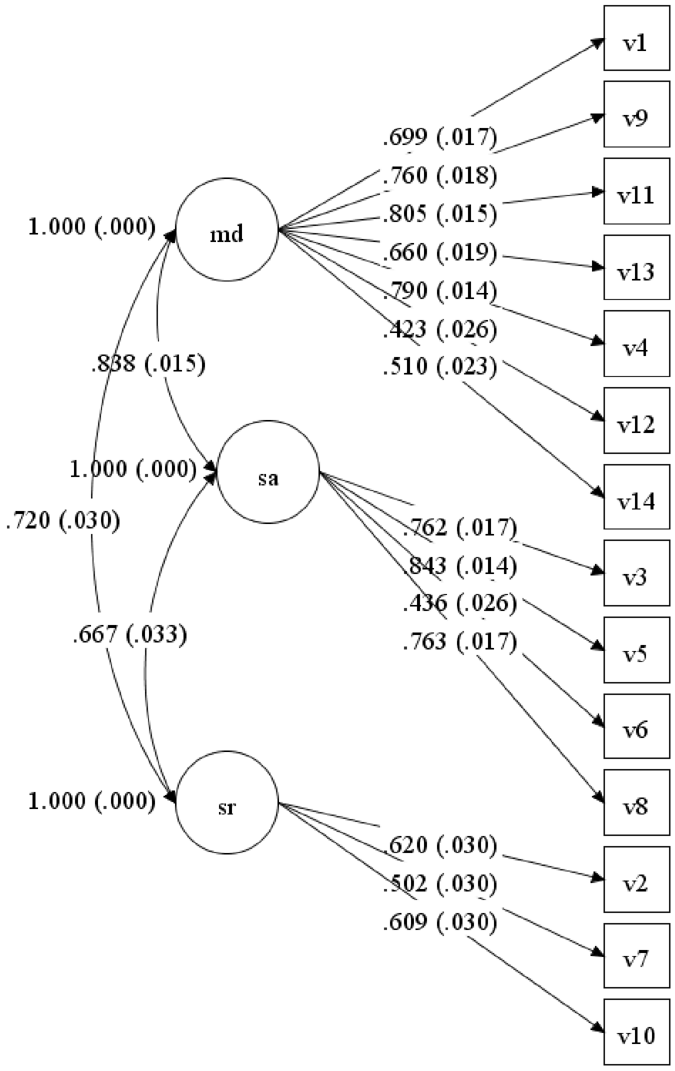


```
Chi-Square Test of Model Fit
          Value                              624.395*
          Degrees of Freedom                      74
          P-Value                             0.0000
RMSEA (Root Mean Square Error Of Approximation)
          Estimate                             0.067
          90 Percent C.I.                      0.062  0.072
          Probability RMSEA <= .05             0.000
CFI/TLI
          CFI                                  0.954
          TLI                                  0.944
Chi-Square Test of Model Fit for the Baseline Model
          Value                            12076.025
          Degrees of Freedom                      91
          P-Value                             0.0000
SRMR (Standardized Root Mean Square Residual)
          Value                                0.044
```

One factor model (Model 3)

```
MODEL FIT INFORMATION
Chi-Square Test of Model Fit
          Value                              896.142*
          Degrees of Freedom                      77
          P-Value                             0.0000
RMSEA (Root Mean Square Error Of Approximation)
          Estimate                             0.080
          90 Percent C.I.                      0.075  0.085
          Probability RMSEA <= .05             0.000
CFI/TLI
          CFI                                  0.932
          TLI                                  0.919
Chi-Square Test of Model Fit for the Baseline Model
          Value                            12076.025
          Degrees of Freedom                      91
          P-Value                             0.0000
SRMR (Standardized Root Mean Square Residual)
          Value                                0.054
```
