# Supplementary figures and images for: Development and Initial Validation of the Russian Version of the RAADS-14: A Self-Report Questionnaire to Assess Autistic Traits
Source: Eur J Investig Health Psychol Educ. 2023 Nov 20;13(11):2724–35. doi: 10.3390/ejihpe13110188 (PMC10670239; doi:10.3390/ejihpe13110188)

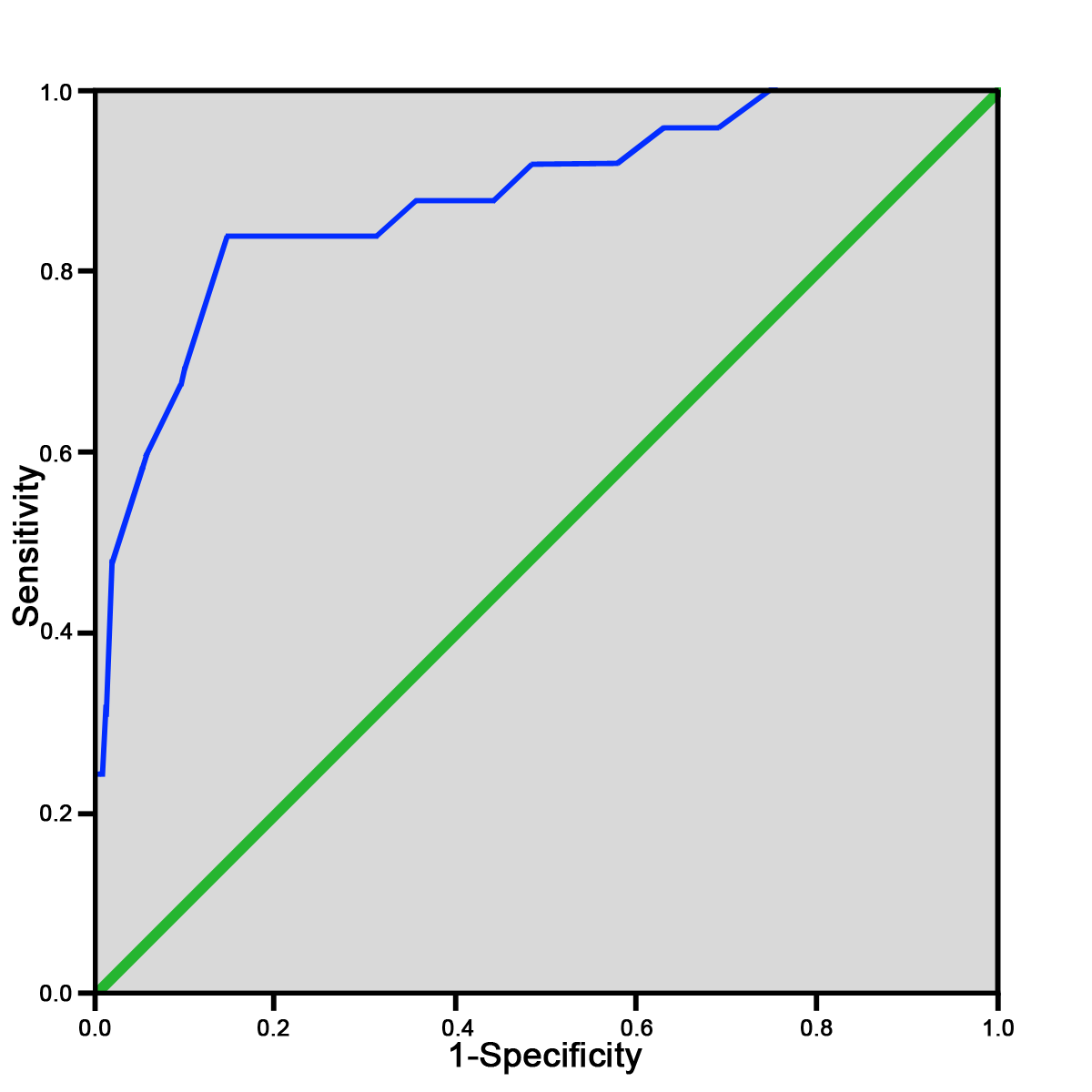

Supplement: Supplementary file 1 [file ejihpe-13-00188-s001.zip › Figure S1. ROC curve.tif]
